# TMS Database Registry Consortium Research Project in Japan (TReC-J) for Future Personalized Psychiatry

**DOI:** 10.3390/jpm12050844

**Published:** 2022-05-22

**Authors:** Yoshihiro Noda, Junichiro Kizaki, Shun Takahashi, Masaru Mimura

**Affiliations:** 1 Department of Neuropsychiatry, School of Medicine, Keio University, Tokyo 160-8582, Japan; mimura@a7.keio.jp; 2 TENNE Inc., Kyoto 604-8241, Japan; j.kizaki@tenne.co.jp; 3 Department of Psychiatry, Graduate School of Medicine, Osaka University, Osaka 565-0871, Japan; s.takahashi@psy.med.osaka-u.ac.jp; 4 Clinical Research and Education Center, Asakayama General Hospital, Osaka 590-0018, Japan; 5 Graduate School of Rehabilitation Science, Osaka Metropolitan University, Osaka 583-8555, Japan; 6 Department of Neuropsychiatry, Wakayama Medical University, Wakayama 641-0012, Japan

**Keywords:** transcranial magnetic stimulation (TMS), treatment-resistant depression (TRD), database, registry, stratified psychiatry, the Japan Society for Clinical TMS Research

## Abstract

The registry project led by the Japanese Society for Clinical Transcranial Magnetic Stimulation (TMS) Research aims to establish a centralized database of epidemiological, clinical, and biological data on TMS therapy for refractory psychiatric disorders, including treatment-resistant depression, as well as to contribute to the elucidation of the therapeutic mechanism of TMS therapy and to the validation of its efficacy by analyzing and evaluating these data in a systematic approach. The objective of this registry project is to collect a wide range of complex data linked to patients with various neuropsychiatric disorders who received TMS therapy throughout Japan, and to make effective use of these data to promote cross-sectional and longitudinal exploratory observational studies. Research utilizing this registry project will be conducted in a multicenter, non-invasive, retrospective, and prospective observational research study design, regardless of the framework of insurance medical care, private practice, or clinical research. Through the establishment of the registry, which aims to make use of data, we will advance the elucidation of treatment mechanisms and identification of predictors of therapeutic response to TMS therapy for refractory psychiatric disorders on a more real-world research basis. Furthermore, as a future vision, we aim to develop novel neuromodulation medical devices, algorithms for predicting treatment efficacy, and digital therapeutics based on the knowledge generated from this TMS registry database.

## 1. Introduction

The World Health Organization (WHO) estimates that there are approximately 300 million patients with depression, 50 million patients with cognitive disorders, 23 million patients with schizophrenia, and 60 million patients with bipolar disorder worldwide [1,2]. The Lancet Commission paper in 2018 reported that the number of mental illnesses, including depression, is increasing worldwide and could cost the global economy up to $16 trillion from 2010 to 2030 if appropriate countermeasures were not taken [3]. It is estimated that some of the costs will be direct, such as health care, pharmaceuticals, and other treatments, while most costs will be indirect, such as productivity losses, social welfare expenditures, education, and law-and-order-related issues. As such, the report stated that a worsening crisis could cause permanent harm to people, societies, and economies on a global scale.

In Japan, the number of patients with mental health problems visiting healthcare providers exceeds 4.2 million (Annual Report on Health, Labor and Welfare in Japan). In particular, approximately 70% of the patients with depression do not achieve remission even after adequate medication, and, in those cases, their symptoms are often persistent [4]. The main factors that have contributed to the ever-increasing number of patients with depression are not only external factors, such as socioeconomic changes, advances in diagnostic technologies, or the population aging, but also the fact that effective treatments to radically cure depression have not yet been sufficiently developed, and more new cases of depression develop than patients who achieve remissions for their depression [5].

While medication, psychotherapy, and electroconvulsive therapy (ECT) are commonly used to treat depression, repetitive transcranial magnetic stimulation therapy (rTMS) has recently emerged as a promising new treatment strategy for medication-resistant depression [6]. The prototype of TMS was developed by Dr. Barker and colleagues in 1985 [7], and subsequent technological innovations and numerous clinical studies have led to the development of various successful TMS treatment protocols [8,9]. The shared therapeutic mechanism of TMS treatment for psychiatric disorders is thought to be normalization through the neuromodulation of neuroplasticity in the prefrontal cortex that is associated with the brain pathology of the respective disorders [10,11]. In fact, brain functions, including the prefrontal cortex, are impaired in various psychiatric disorders, such as depression, autism spectrum disorders, schizophrenia, and mild cognitive impairment, providing support for the therapeutic mechanism of conventional ECT [12], which delivers electrical stimulation to the frontotemporal region. On the other hand, TMS therapy differs from ECT in that it directly stimulates only the localized target brain region that can non-invasively stimulate the least necessary brain regions [13] without general anesthesia or convulsive induction [14].

Standard TMS therapy for treatment-resistant depression, which has the highest level of evidence, achieves a treatment response rate of approximately 40 to 50% [15]. However, conventional standard TMS therapy is limited by its lengthy treatment time, making it difficult to apply in real-world clinical practice [16], as well as its ineffectiveness in severe depression [17]. Moreover, the optimal therapeutic parameters of TMS therapy for depression are currently still in the exploratory phase. This is due to the extremely high biological heterogeneity of the psychiatric disorders being treated, including depression [18], and the myriad combinations of physical time-space parameters that can be employed as therapeutic strategies in TMS therapy [19,20]. In addition, with the rapid advances in data science over the past few years, it has become possible to extract statistical features and classify complex data into patterns by applying modern machine learning and multivariate analysis to multidimensional datasets [21,22,23,24]. Given this background, it is clinically important to collect background information, assessment data before, during, and after TMS therapy, and treatment parameters of patients who receive TMS therapy, and to analyze these data to determine whether it is possible to identify predictors of treatment response to TMS therapy that could lead to the development of therapeutic algorithms of TMS. If such an algorithm for predicting treatment efficacy can be developed, it would enable the setting of optimal treatment parameters for each individual patient and the implementation of more effective TMS therapy for psychiatric disorders, which is of great medical and social significance.

Therefore, this research project on TMS therapy aims to collect clinical epidemiological information, clinical course, and TMS treatment parameters for patients undergoing TMS therapy and to establish a patient database registry related to TMS therapy so that, in the future, predictors of the treatment response to TMS therapy can be identified and optimal treatment parameters can be configured according to individual patients’ data. Furthermore, the future goal is to develop algorithms for predicting treatment efficacy through comprehensive, exhaustive, and exploratory data analysis of the TMS database registry.

## 2. The TReC-J Project Execution Protocol

### 2.1. Study Subjects and Target Psychiatric Disorders

Patients with any psychiatric disorders who have received or will receive TMS therapy, regardless of whether it is covered by public medical insurance, private practice, or clinical research, can be included. Specifically, the majority of the data to be registered in the TReC-J project will be data on patients with a diagnosis of major depressive disorder, but, since it will also include clinical research data and real-world data from private practice other than public medical insurance, it may include a certain number of cases with a primary diagnosis of other psychiatric disorders besides major depressive disorder, such as bipolar depression, anxiety disorders, secondary depression associated with neurodevelopmental disorders (e.g., autistic spectrum disorder or attention deficit hyperactivity disorder), depression associated with adjustment disorders, and depression associated with schizophrenia. However, the diagnosis of these psychiatric disorders is based on the diagnostic criteria defined in the DSM-5.

Of note, this TMS database registry project, entitled “Research on TMS therapy related database registry construction”, was centrally and collectively reviewed by the Kyoto University Graduate School and Faculty of Medicine Ethics Committee and was initially approved on 24 June 2021.

### 2.2. Eligibility Criteria

Study subjects who meet all of the following inclusion criteria and none of the exclusion criteria are eligible for registration in this project. The eligibility criteria for subject data in this project are in compliance with the IFCN Expert Guidelines [25].

#### 2.2.1. Inclusion Criteria

(1) Receiving or planning to receive TMS therapy, regardless of whether it is covered by health insurance, private practice, or clinical research.
(2) Age at the time of consent is between 18 and 90 years old.
(3) Written consent to participate in the registry has been obtained from the research subject or a substitute. Consent by a substitute should be obtained only if the research subject is a minor or has a significant cognitive impairment. However, for patients who have received TMS treatment in the past and cannot be contacted for informed consent, the data may be used by making the opt-out document available on the website or hospital notice board during the study period, thereby giving them an opportunity to refuse secondary use of the data. In this case, “age at the time of obtaining consent” in inclusion criterion (2) is to be read as “age at start of TMS treatment”.

#### 2.2.2. Exclusion Criteria

(1) Those whom the investigator considers unable to complete the scheduled treatment prior to the start of TMS therapy.
(2) Those for whom the investigator determines, prior to the introduction of the TMS therapy, that it is not effective for the subject in the first place.
(3) Patients with any of the following complications:
  – Organic brain disease (e.g., intracranial organic lesions of moderate severity or higher, etc.).
  – Substance-related disorders in the 6 months prior to the start of TMS treatment.
  – Serious or unstable physical conditions.
(4) Patients with a history of any of the following:
  – Convulsive seizures or epilepsy (except febrile convulsions in childhood).
(5) Patients with metal implants in the head and neck (e.g., metal implants, pacemakers, etc.).
(6) Patients who are deemed inappropriate as research subjects for data collection in this registry by the principal investigator or co-investigators.

### 2.3. Research Design

**Figure 1:**
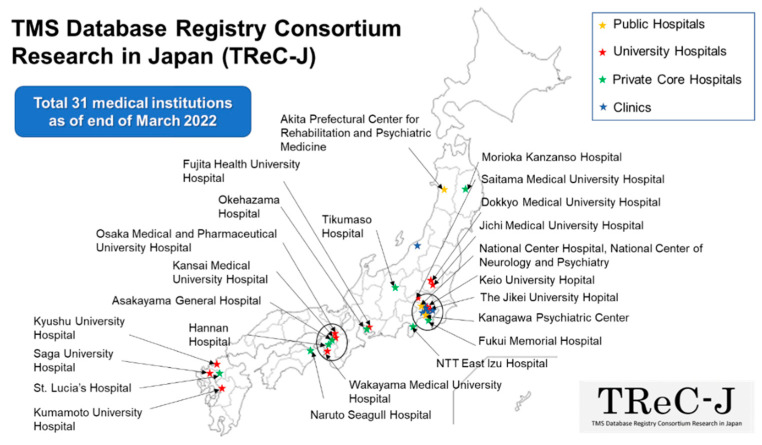
**TMS Database Registry Consortium Research in Japan (TReC-J).** As of March 2022, a total of 31 medical institutions throughout Japan are participating in this registry project. As marked by the black circles on the map, TMS facilities in Japan are concentrated in the Tokyo metropolitan area and in the Kansai area centered in Osaka.

This registry project will be conducted within the framework of the multicenter, non-invasive, retrospective, and prospective observational studies (see Figure 1).

### 2.4. Equipment to Be Used

The types of medical devices used and other information will also be collected in the database registry, but this registry does not limit the type of equipment used.

### 2.5. Registration Procedures

Registration of research subjects will be conducted using an electronic data capture (EDC) system. After obtaining consent from research subjects, the principal investigators and co-investigators will confirm that there is no problem with the inclusion and exclusion criteria with respect to the subject(s). A subject identification code is then assigned by registering basic information that meets eligibility criteria in the EDC. The research subject code is transcribed and stored in the anonymization correspondence table, together with information that can identify the research subject. No personally identifiable information will be entered into the EDC itself.

### 2.6. Observed Items, Examined Data Items, and Reported Items

The following are examples of data items collected in this registry study, along with the approximate timing of data acquisition. If there are data before consent is obtained, they will be collected with the consent of the research subject, if possible. In addition, each investigator is required to specify the type of TMS equipment employed and the details of the treatment protocols in the reported items, respectively.

**Figure 2:**
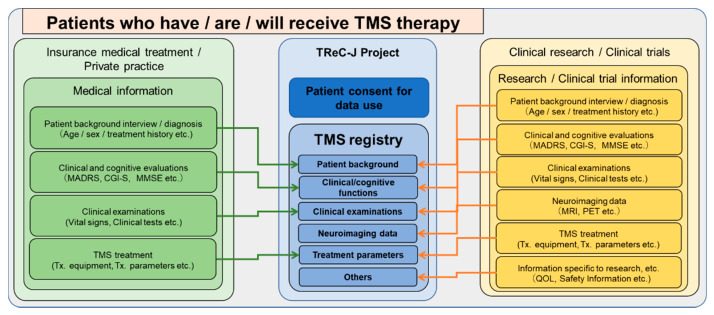
**The general framework of the various data attributes and datasets stored in the TMS Registry.** This TMS registry is designed to allow the registration of TMS-therapy-related data in both prospective and retrospective fashion within a variety of frameworks, such as insurance medical care, private practice, clinical research, and post-marketing surveillance.

These data will be obtained through the usual medical care (insurance covered or private practice) or clinical research when TMS therapy is performed and will not be newly obtained solely for the purpose of this registry study (see Figure 2). However, if there is information (e.g., questionnaires) that is obtained separately at the discretion of the physician in charge within the range of usual medical care at the participating institutions in this registry study, an appropriate explanation should be provided to the research subjects that such information is to be obtained within the range of usual medical care.

### 2.7. Evaluation Items as Endpoints

It is envisioned that the background (e.g., treatment history), pathological conditions, and various evaluations and treatment parameters performed on patients receiving TMS therapy will be collected in this registry database to seek background factors and treatment parameters associated with treatment efficacy. However, at this time, the registry is not a database dedicated solely to specific endpoints, and general information and examination results are subject to collection and storage. If new endpoints other than those currently envisioned are added during the course of this registry study, we plan to revise the research protocol and have it reviewed again by the Ethics Committee.

### 2.8. Statistical Analysis

Population to be analyzed: The primary purpose of this registry study is to collect data for the construction of a database on TMS therapy in Japan. For this reason, we did not set a specific statistical design for this registry study, which was planned from the beginning. In addition, due to the nature of the registry, the target population for analysis is expected to change from time to time depending on the purpose of the study, and, thus, no specific target population for analysis has been set at this time.

Primary analysis: This registry study aims to comprehensively collect data on patients receiving TMS therapy for the purpose of constructing a database on TMS therapy in Japan, with no specific primary or secondary endpoints at this time. In the database construction phase, the median and standard deviation will be calculated for continuous data for the tabulation of each collection item. For binary and categorical data, percentages (%) will be calculated. The mean and standard deviation are used for the aggregation of scores for clinical evaluations, which are important measures in TMS therapy.

Secondary analysis: To assess the safety of TMS therapy, the collected adverse events and their rates will be summarized by TMS device used or TMS protocol employed.

Target number of enrolled cases: All data from study subjects who meet eligibility and do not violate the inclusion/exclusion criteria may be enrolled. The initial target number of cases to be enrolled is 1000. Here, as the scale of the TReC-J project expands and its duration is extended, this target number of data will also increase. Since the purpose of this study is to collect data for the construction of the TMS therapy database, we will collect as much data related to TMS therapy as possible. In order to prevent inappropriate data that may become noise from being registered in the registry database as much as possible, it is our policy to exclude in advance any target data that do not correspond to the purpose and inclusion criteria of this registry project.

### 2.9. Data Management

For the preparation and output of case report forms, this registry project will operate using an EDC system, called the “Personal Monitoring System,” that we developed for this project. The collaborating institutions will separately and appropriately store primary data as source documents, which will be the source of data to be entered into the EDC system. The data provided by subjects in paper form as source documents will be reviewed at each collaborating institution, and each research collaborator will responsibly manage and store those records. The principal investigator, co-principal investigators, and their research assistants shall enter the data required in the protocol into the electronic case report form in the EDC system. In addition, the case report form will be prepared and the data entered will be changed or modified in accordance with the case report form entry rules. The principal investigator and co-principal investigators are responsible for approving and assuring that the data entered are complete and accurate.

**Figure 3:**
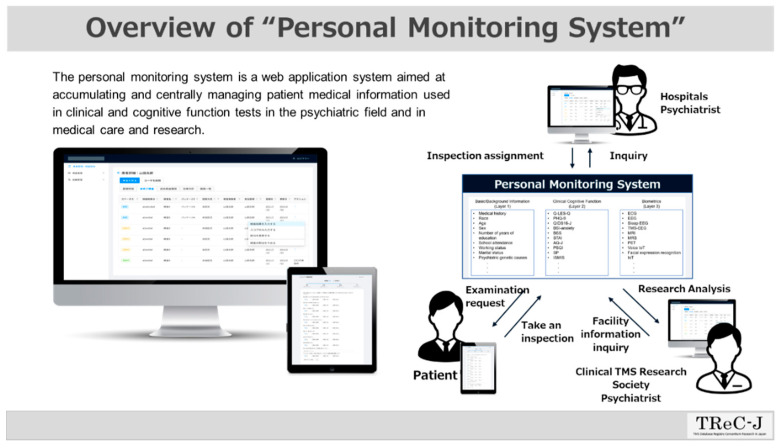
**Overview of the Personal Monitoring System.** We have developed a unique application software for personal monitoring dedicated to this TMS registry, and have constructed and implemented a system that enables data input and the confirmation and approval of examination results via PC and tablet terminals at each of the medical institutions collaborating in this project.

Case report data in this registry study will be stored as an electronic database via the EDC system during the study period. After the final data entry and correction are completed, copies of the case report form data (including correction records) will be appropriately stored at each collaborating institution until the storage period stipulated in this study. The results of questionnaires and self-administered rating scales for the study subjects can also be entered into this registry database by the study subjects themselves using individual terminals (see Figure 3).

### 2.10. Data Handling

If any problem arises with the registration data, the principal investigator and the statistical analyst will discuss with each other and decide how to manage the data, and the items, contents, and date of the decision will be recorded.

### 2.11. Methods and Operating Systems for Storing Clinical Epidemiological Information, Psychometric Data, and Biological Data of Subjects in This Registry Database

**Figure 4:**
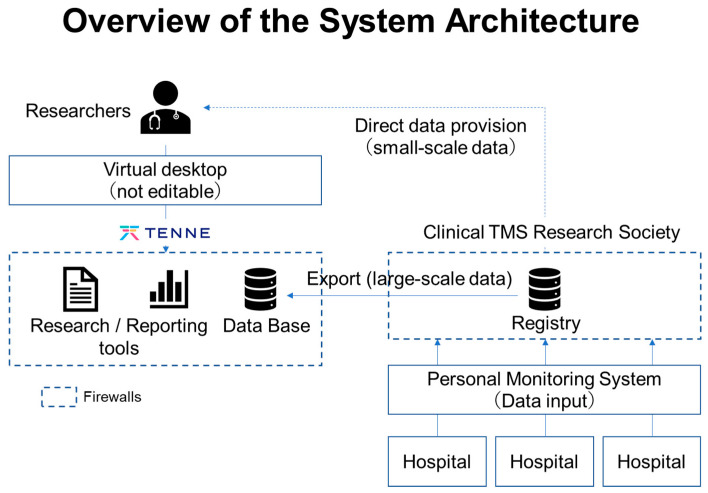
**Overview of the System Architecture.** TMS-therapy-related data collected from each collaborating medical institution via the Personal Monitoring Systems will be anonymized and stored in a cloud-based database managed by the Japan Society for Clinical TMS Research. In addition, a partner company, TENNE Inc., will handle the system maintenance and operational management.

The objective of this study is to establish a database related to TMS therapy. Thus, long-term storage is necessary for comprehensive, exhaustive, and exploratory analyses of the data collected in this database. The TMS registry database will be managed by the Japan Society for Clinical TMS Research on a cloud, and part of its operation will be outsourced to a partner company, TENNE Inc. Specifically, the Personal Monitoring System in the TReC-J project employs the Google Cloud Platform, a robust cloud certified under ISO/IEC27001. Furthermore, the system development company constantly monitors the data storage status to ensure the security of the system, including data protection and error handling. In addition, the Personal Monitoring System described above has a function to automatically and directly store the self-administered data entered by the subjects themselves and the clinical and psychological evaluation data obtained by the examiner after interviewing the subjects in an anonymized form into the database (see Figure 4). Thus, the database will contain the information of subjects in a completely anonymized form.

Moreover, the TReC-J project is developing a database platform that conforms to domestic agreements of the Act on the Protection of Personal Information, Guidelines for the Use of Cloud Services for Medical Institutions by the Ministry of Health, Labour and Welfare, the Ministry of Internal Affairs and Communications, and the Ministry of Economy, Trade and Industry in Japan. Furthermore, a partner company of this TReC-J project, TENNE Inc., owns a software license developed by BC Platforms, which is compliant with international standards, such as the General Data Protection Regulation (GDPR) and the Health Insurance Portability and Accountability Act (HIPPA), and can implement a virtual analysis environment. Therefore, when conducting international collaborative research projects, it is possible to build virtual data storage environments at domestic and international collaborating research institutions and consolidate them for analysis. In addition, the Personalized Monitoring System used in this TMS registry project also includes functions for simple logical checks for data cleaning, change history management, and output of data forms. Furthermore, the local servers and cloud databases used in this registry have robust security systems that meet quality control (computerized system validation) and electromagnetic record reliability (electronic record / electronic signature guidelines) requirements.

**Figure 5:**
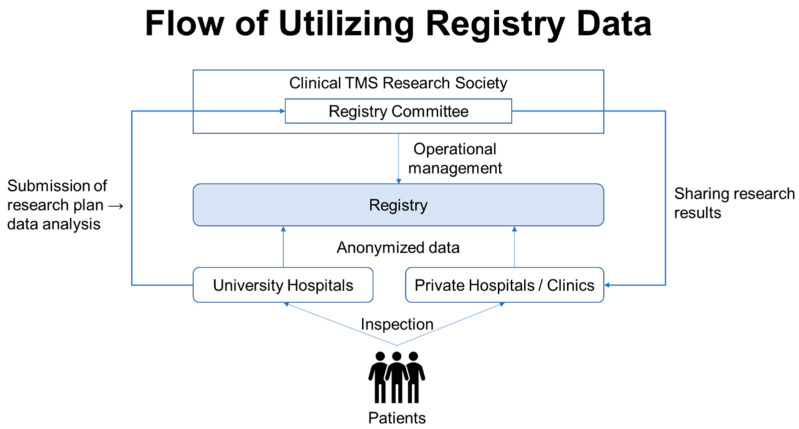
**Flowchart diagram of the application and approval process for using the TMS registry data.** The use of TMS registry data is limited to the members of the Japan Society for Clinical TMS Research, who must first submit a proposal to the society regarding the purpose of use and analysis. The registry data will be made available to the researchers upon approval of the proposal by the registry data use review committee established within the Japanese Society for Clinical TMS Research.

Note that, from the standpoint of maintaining the confidentiality of subjects, hospital-managed medical records and other medical data, records of subjects’ consent, a list of correspondence between subjects’ personal information and subjects’ identification codes, and case report forms and similar documents will be stored securely in locked cabinets at each collaborating research institution. Furthermore, the use of the data collected in this registry study will be reviewed by the registry data use review committee organized by the Japan Society for Clinical TMS Research, which will review the purpose and scope of each study, and upon approval, the anonymized data for analysis will be provided to the parties involved in the collaborative research institutions of this project. Thereafter, the data will be used for secondary purposes, such as elucidating the therapeutic mechanism of TMS and determining the therapeutic efficacy of TMS (see Figure 5).

In addition, in the future, there are possibilities that a portion of the registry data will be provided to medical device manufacturers and pharmaceutical companies for secondary use for commercial purposes, such as the development of TMS therapy technologies. The possibility of such secondary use will be clearly stated in the informed consent form, and consent will be obtained from the subjects in principle.

When using this registry data for analytical research or providing registry data to a third party, it is necessary to separately obtain approval from the Ethics Review Committee, or at least the registry data use review committee in the Japan Society for Clinical TMS Research. Moreover, when a new purpose for the use of registry data is identified, information on the specific purpose of the data use will be made available to the subjects in an opt-out document. The research personnel will then contact the subject whenever possible to provide oral explanations and to ensure that the subject has the opportunity to withdraw consent for the applicable analytic studies to be conducted with the subject’s data. The principal investigator takes responsibility for managing the information collected in this registry project.

### 2.12. Method of Information Disclosure Regarding this Registry Study

A summary of this study is available on the Japan Registry of Clinical Trials (jRCT), a database operated by the Ministry of Health, Labour and Welfare (MHLW), as follows: jRCT1050210059; 5 August 2021. The published information will be updated as necessary according to the revision of the research protocol and the progress of the study. When presenting the results obtained through this registry research in an academic paper or at a conference, the reporter should consult and confirm the appropriateness of such presentation with the principal investigator, registry committee members, and, if applicable, the respective co-investigators, as appropriate.

### 2.13. The Registry Project Period

Research data registration period is 24 June 2021–31 March 2025 (with possible extensions as appropriate), and study period is 24 June 2021–31 March 2026 (may be extended as needed). The subjects will be followed for a period of one year, starting from the date of completion of TMS therapy, to the extent possible. If the consent is obtained from the subject, follow-up will be conducted as long as possible thereafter. Note that the TMS therapy database is a study that is intended to be operated on a permanent basis. Therefore, a notification of further study extension will be submitted at the end of the five-year study period.

## 3. Discussion

**Figure 6:**
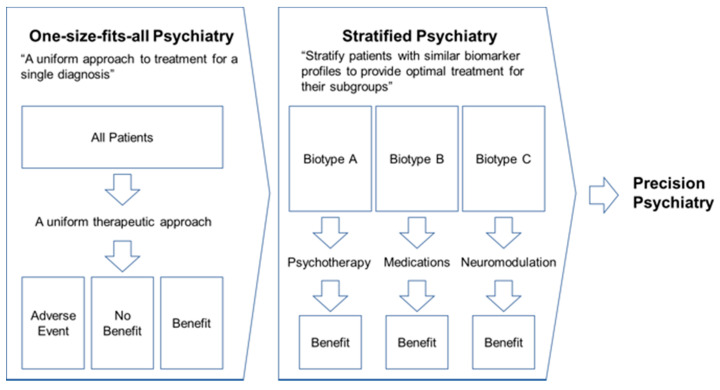
**A paradigm shift from “One-size-fits-all Psychiatry” to “Stratified Psychiatry”.** The approach required for the future of psychiatry is not “a uniform treatment for a specific diagnosis” but “redefinition of diseases based on biotypes related to each diagnosis and development of effective treatments for each biotype”. To realize this goal, it is essential to establish the TMS registry as proposed in this protocol and commission paper.

As of March 2022, a total of 31 collaborating institutions throughout Japan are participating in this registry project and diligently accumulating TMS therapy-related data. The diagnosis and treatment optimization of psychiatric disorders require a wide range of information and knowledge, such as patient biometric and behavioral data, in addition to the usual clinical data. In recent years, it is expected that objective and quantitative interpretation and diagnosis of psychiatric disorders will be implemented by utilizing real-world data [26]. In particular, psychiatry in the first half of the 21st century calls for a paradigm shift from “one-size-fits-all psychiatry”, a uniform psychiatric model, to “stratified psychiatry”, a redefinition of psychiatric disorders based on their biotypes [27] (see Figure 6). In Japan, TMS therapy for treatment-resistant depression has been covered by public medical insurance since 2019, which has resulted in a gradual spread throughout the country. To date, the clinical and epidemiological data related to TMS therapy in Japan have been scattered across various medical institutions. As a result, there has been little collaboration in the sharing of such data, and, thus, uniform and sustainable data acquisition has not been achieved yet. Therefore, it is necessary to develop and promote the TMS database registry that can consolidate clinical and biological data, as well as epidemiological information, to establish a platform for future research and development on TMS therapies for psychiatry disorders.

Currently, a number of collaborative research sites from all over Japan are participating in the TMS database registry project, and an all-Japan project is being initiated to enable the first big data analysis related to TMS therapy. This registry project will also develop a feedback loop to provide the results of the analysis to the data providers (each principal investigator and each subject). Such services will also enable the collection of use cases for clinical and healthcare applications and data utilization in the area of psychiatric disorders, such as early detection and intervention to prevent the onset of mood disorders and to initiate treatment at a milder stage of symptoms.

Furthermore, as a future prospect, after the database registry project in Japan gets on track, we would like to expand and develop this registry project to include not only domestic but also foreign collaborating research institutes to enable mutual data sharing under certain conditions.

## Data Availability

The dataset constructed and consolidated in this registry project may be made available by the principal investigator if the content and purpose of the analysis proposed by the members of the Japan Society for Clinical TMS Research are appropriate and reasonable after review and approval by the registry committee established by the research society.
